# Effects of Dexmedetomidine on Postoperative Nausea and Vomiting in Adult Patients Undergoing Ambulatory Thyroidectomy: A Randomized Clinical Trial

**DOI:** 10.3389/fmed.2021.781689

**Published:** 2021-12-13

**Authors:** Cuiyu Xie, Caihui Zhang, Hao Sun, Yao Lu

**Affiliations:** ^1^Department of Anesthesiology, The First Affiliated Hospital of Anhui Medical University, Hefei, China; ^2^Ambulatory Surgery Center, The First Affiliated Hospital of Anhui Medical University, Hefei, China

**Keywords:** dexmedetomidine, postoperative nausea and vomiting, ambulatory, thyroidectomy, anesthesia

## Abstract

**Background:** Postoperative nausea and vomiting (PONV) is a common and disturbing problem in patients undergoing ambulatory thyroidectomy. This prospective trial aimed to explore whether dexmedetomidine (DEX) combined with azasetron (AZA) can further drop the incidence of PONV in patients undergoing ambulatory thyroidectomy compared with AZA.

**Methods:** This single-center, randomized, double-blind trial involved 172 adult patients undergoing ambulatory thyroidectomy. The individuals were randomized to DEX + AZA group and AZA group. In the DEX + AZA group, patients received dexmedetomidine 0.5 μg kg^−1^ for 10 min and then the infusion rate was held at 0.1 μg kg^−1^ h^−1^ until the completion of the operation, while the same amount of 0.9% saline in the AZA group. At the completion of the surgery, 10 mg azasetron was administered to every patient in both groups. The primary outcome was the incidence of 24 h PONV after ambulatory thyroidectomy. The secondary outcomes included residence time in recovery room, pain scores, severity of nausea, and adverse events.

**Results:** No significant difference was found in the incidence of 24-h PONV between the DEX + AZA group and the AZA group [36% (30 of 84) vs. 38% (32 of 84); relative risk, 0.94; 95% confidence interval (CI), 0.63–1.40; *P* = 0.749]. The incidence of severe nausea was similar between the DEX + AZA group and the AZA group [57% (12 of 21) vs. 43% (9 of 21); relative risk, 1.33; 95% CI, 0.72–2.50; *P* = 0.355].

**Conclusions:** Intraoperative dexmedetomidine combined with azasetron failed to drop the incidence of 24-h PONV compared with azasetron alone in patients undergoing ambulatory thyroidectomy.

## Introduction

At present, thyroid tumors and thyroid disease are on the rise worldwide. In China, people with thyroid tumors are increasing rapidly, and the incidence rate is 3 times more common in women than in men (1, 2). Surgery is the main treatment for thyroid cancer. Guided by the concept of enhanced recovery after surgery, ambulatory thyroidectomy has become a new trend, and more and more thyroidectomies have been performed in the ambulatory operating room (3, 4).

Postoperative nausea and vomiting (PONV) refer to any nausea, retching, and vomiting that happen after the anesthesia and operation (5). Studies have reported that, if not treated, ~35% of patients will develop nausea, vomiting, or both symptoms after surgery, which is a very painful experience, even more serious than postoperative pain (6). However, ~60–80% of patients develop nausea and vomiting after thyroidectomy without antiemetic prophylaxis, which may result in severe complications, such as aspiration pneumonia caused by accidental inhalation of vomitus or postoperative neck hematoma and even airway obstruction caused by hematoma (7). PONV often prolongs the patient's stay in the recovery room or even the discharge time, which is the main reason for accidental readmission after elective surgery (8). However, prophylactic antiemetic agents can improve the condition.

The latest guidelines for the management of PONV advised that the combination of different prophylactic drugs can drop the rate of PONV (9). Dexmedetomidine (DEX) is a novel α2-adrenoreceptor agonist that has good sedative, analgesic, and sympatholytic effects. Because of its advantages of reducing intraoperative opioid dose and accelerating postoperative recovery, dexmedetomidine is increasingly used during anesthesia. Recently, several researches have suggested that intraoperative DEX may have an antiemetic effect and can decrease the incidence of PONV (10, 11). Azasetron (AZA) as a selective serotonin receptor antagonist that is recommended by the guidelines as a first-line antiemetic agent in high-risk patients. However, there have been no studies of dexmedetomidine in combination with azasetron for the prevention of PONV after ambulatory thyroidectomy.

We designed this trial to explore the effect of dexmedetomidine on nausea and vomiting after ambulatory thyroidectomy. We hypothesized that dexmedetomidine combined with azasetron can further decrease the incidence of nausea and vomiting 24 h after ambulatory thyroidectomy compared with azasetron.

## Methods

### Ethics

Ethical approval for this study (PJ2020-13-21) was provided by the Ethics Committee of The First Affiliated Hospital of Anhui Medical University, Hefei China (Chairperson Prof Heng Wang) on 29 October 2020. This randomized, double-blind trial was registered at the Chinese Clinical Trial Registry (ChiCTR2000039603, Principal Investigator: Yao Lu, MD, PhD, November 03, 2020). Individuals scheduled to undergo ambulatory thyroidectomy were enrolled. An informed consent was obtained from each patient.

### Study Design

Eligible patients were 18–60 years old of any sex with American Society of Anaesthesiologists classification I–II and body mass index of 18–30 kg/m^2^. The exclusion criteria included allergy to dexmedetomidine, antiemetic or analgesic medication intake within 24 h before surgery, use of antipsychotic drugs or corticosteroids, previous heart failure or arrhythmia and diabetes mellitus with poor glycemia control, uncontrolled hypertension, liver and kidney dysfunction, gastric dynamic disorder, and vestibular disease.

### Randomization and Masking

The investigators, independent of the blind study, prepared the randomized schedule and study drugs. During the study, patients, investigators, anaesthesiologists, nurses in the post-anesthesia care unit (PACU) or ward, and the recorders of each observation index were all unaware of the group assignment. The SPSS version 23.0 generated random numbers, which were delivered in sealed, opaque envelopes. And the envelopes were opened after consent was obtained. The study drugs included dexmedetomidine (100 μg ml^−1^, diluted with 0.9% saline to 4 μg ml^−1^), azasetron, and 0.9% saline, all prepared according to random numbers. Patients were randomly allocated into the DEX + AZA group or the AZA group in a 1:1 ratio.

### Study Treatments

The patient received routine pre-operative preparation, and no premedication was administered. Standard monitoring was done after arriving at the operating room. Before anesthesia induction, 0.5 μg kg^−1^ for 10 min of dexmedetomidine was administered intravenously and then the infusion rate reduced to 0.1 μg kg^−1^ h^−1^ until the end of surgery in the DEX + AZA group or the same amount of 0.9% saline in the AZA group. General anesthesia was induced i.v. with propofol (2 mg kg^−1^) and sufentanil (0.4 μg kg^−1^). To facilitate endotracheal intubation, cisatracurium (0.2 mg kg^−1^) was administered. Anesthesia was maintained with remifentanil (0.1–0.2 μg kg^−1^ min^−1^) and propofol (4–6 mg kg^−1^ h^−1^), with their infusion rate adjusted to keep the bispectral index from 40 to 60. Sufentanil (5–10 μg) was administered to keep the heart rate and blood pressure fluctuations within 20% of baseline. In addition, to maintain adequate muscle relaxation, intermittent doses of cisatracurium were administered. At the end of surgery, every patient in each group was treated with 10 mg azasetron and 50 mg flurbiprofen axetil. Hypotension (mean arterial pressure of <60 mmHg or <80% of the baseline) was treated with 6 mg ephedrine and bradycardia (heart rate of <45 beats min^−1^) with atropine 0.5 mg. After the operation, when autonomous breathing was regular, tidal volume was >6 mL kg^−1^, SpO2 could be maintained at more than 95% during air intake, the trachea catheter was withdrawn, patients were transported to PACU. No analgesia was routinely applied.

### Outcomes

Data were collected by investigators who were unaware of randomization and did not participate in the whole procedure. Relevant baseline characteristics and perioperative data of patients were carefully recorded.

The primary outcome was the incidence of 24 h PONV after ambulatory thyroidectomy. PONV was defined as at least 1 incident of nausea, vomiting, or retching or a combination of these symptoms. The investigators assessed the incidence of PONV at the time of leaving PACU, 6 and 24 h postoperatively in the ambulatory surgical ward by asking patients if they had experienced nausea and vomiting. Only a “YES” or “NO” answer can be accepted.

The secondary outcomes included severity of nausea, residence time in PACU, visual analog scale (VAS) pain scores at the time of leaving PACU, 6 and 24 h postoperatively, and adverse events.

Moreover, 11-point numerical rating scale (NRS) was used to assess nausea severity (0, no nausea; 10, worst nausea imaginable). Mild nausea is defined as NRS score from 1 to 3, whereas severe nausea is >3 (12). Rescue antiemetic agents will be administered if patients have experienced severe nausea or 2 or more times of vomiting or retching or patients request an antiemetic agent at any time. Intramuscular injection of 10 mg metoclopramide hydrochloride was used as the rescue antiemetic agent. Morphine is not allowed to be used after surgery. Parecoxib sodium 40 mg will be administered for VAS pain score of >3. In the PACU, Ramsay sedation scale (RSS) was used to assess sedation levels (1, agitated; 2, cooperative and oriented; 3, can respond to simple questions; 4, asleep, but with a quick reaction to stimulus; 5, asleep, arousable; 6, asleep, unarousable) (13). Oversedation was defined as RSS value of >4.

### Statistical Analysis

Based on our unpublished pilot trial, we observed that, despite 10 mg azasetron being given intraoperatively, ~40% of patients develop PONV after thyroidectomy. A 20% decline in the occurrence of PONV is considered clinically significant; hence, 80 patients are required per group with an alpha level of 0.05 and power of 80%. Finally, we planned to enroll 196 patients to allow for a 20% dropout. Categorical data are presented as number (percentage) and were analyzed by χ^2^ test or Fisher's exact test. Independent *t*-test or Mann-Whitney *U*-test was applied for analyzing continuous variables, which are presented as mean ± standard deviation or medians (interquartile range). χ^2^ test or Fisher's exact test was used to analyze the incidences of PONV, nausea severity, and adverse events presented as number (percentage). Postoperative pain scores are presented as median (interquartile range) and were analyzed with the linear mixed model. Two-tailed *P* < 0.05 was considered statistically significant. SPSS 23.0 was used for data analysis.

## Results

**Figure 1:**
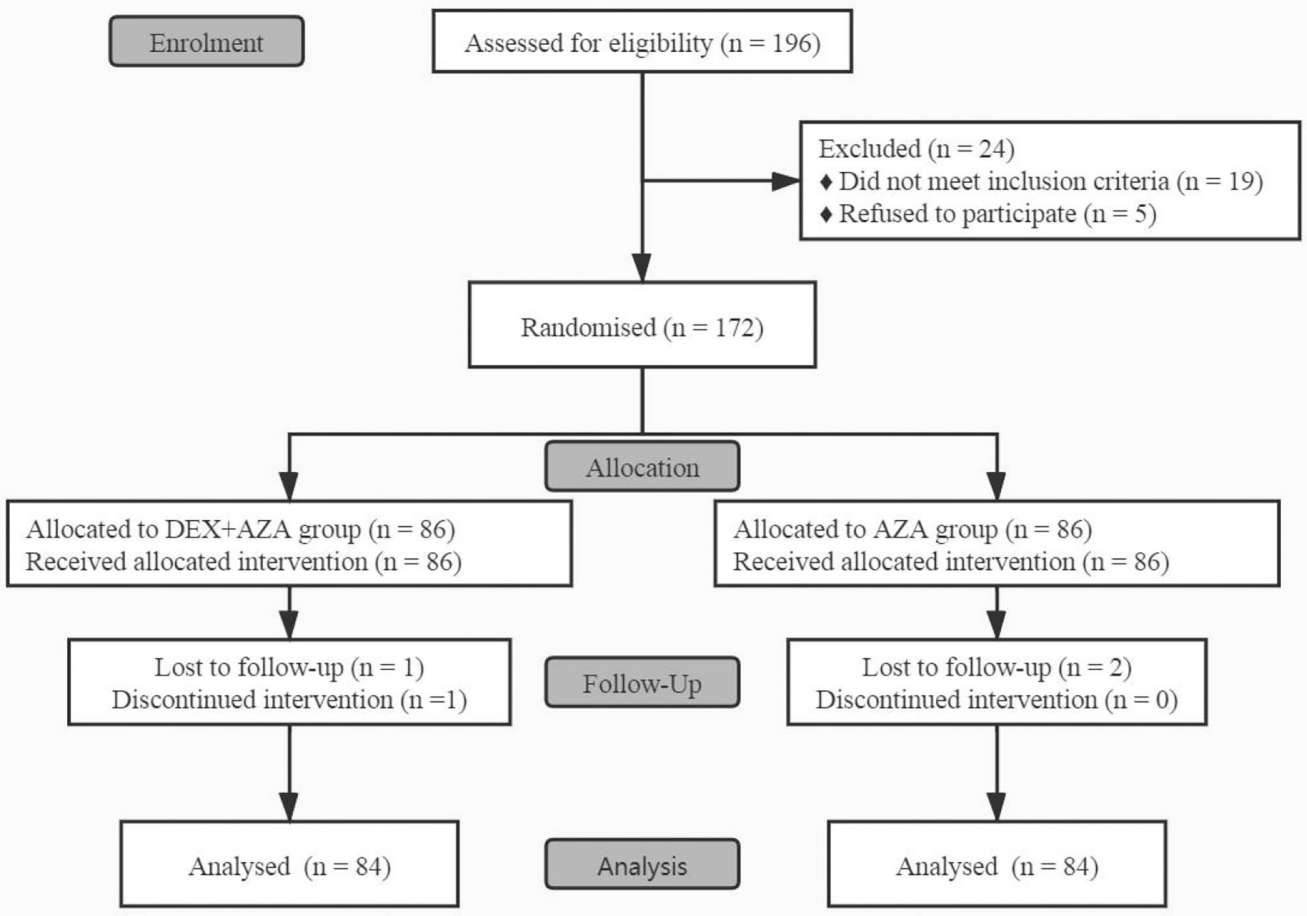
CONSORT flow diagram for this study. This flow diagram shows this single-center, double-blind, randomized trial conducted on adult patients undergoing ambulatory thyroidectomy from November 2020 to January 2021. In the DEX + AZA group, patients received dexmedetomidine 0.5 μg kg^−1^ for 10 min before anesthesia, and then the infusion rate was held at 0.1 μg kg^−1^ h^−1^ until the completion of operation. In the AZA group, patients received an equal volume of saline with same pattern as the DEX + AZA group. Patients in both groups were given 10 mg azasetron before the end of surgery. DEX + AZA, dexmedetomidine + azasetron; AZA, azasetron.

Before the study began, 24 patients were excluded because they did not meet the inclusion criteria or withdrew their consent, so we enrolled a total of 172 patients between November 2020 to January 2021 (Figure 1). In the DEX+AZA group, 1 patient was excluded from the analysis because of failure to pump dexmedetomidine at the beginning of surgery. Because of missing data, 2 patients in each group were excluded from the analysis after being treated with study drugs. In total, 168 patients finished the study and were evaluated for all study outcomes: 84 in the DEX + AZA group and 84 in the AZA group.

No significant differences were found in baseline characteristics, smoking status, or history of motion sickness between two the groups. No significant differences were found in intraoperative variables between groups except the consumption of remifentanil (Table 1).

**Table 1:** Patient baseline characteristics and intraoperative variables.

**Characteristics/intraoperative variables**	**DEX + AZA (*n* = 84)**	**AZA (*n* = 84)**	***P*** **-value**
Mean age ± SD, yr	44 ± 11	41 ± 10	0.157^a^
Sex, no. (%)			0.717^b^
Female	63 (75)	65 (77)	
Male	21 (25)	19 (23)	
Mean BMI ± SD, kg/m^2^	23.5 ± 2.6	23.2 ± 2.7	0.432^a^
ASA classification, no. (%)			0.070^b^
I	25 (30)	15 (18)	
II	59 (70)	69 (82)	
Smoking, no. (%)	7 (8)	5 (6)	0.549^b^
Hypertension, no. (%)	16 (19)	9 (11)	0.129^b^
History of motion sickness, no. (%)	34 (40)	23 (27)	0.073^b^
Median duration of anesthesia (IQR), min	97 (84, 117)	105 (85, 128)	0.059^c^
Median duration of surgery (IQR), min	79 (67, 98)	89 (72, 110)	0.100^c^
**Medication administered during surgery**			
Median propofol (IQR), mg	595 (500, 675)	558 (482, 656)	0.553
Median remifentanil (IQR), μg	580 (466, 700)	768 (600, 1,019)	<0.001
Median sufentanil (IQR), μg	35 (30, 35)	35 (30, 35)	0.497

*DEX, dexmedetomidine; AZA, azasetron; IQR, interquartile range; ASA, American Society of Anesthesiologists; BMI, body mass index.*

a *Analyzed using Independent-sample t test.*

b *Analyzed using χ^2^ test.*

c *Analyzed using Mann–Whitney U-test*.

### Outcomes

The incidence of 24-h PONV was not significantly different between the two groups (Table 2). In the DEX+AZA group, 30 of 84 patients (36%) experienced at least 1 episode of retching, vomiting, or both during the first 24-h postoperative period compared with 32 of 84 patients (38%) in the AZA group (relative risk, 0.94; 95% CI, 0.63–1.40; *P* = 0.749). The postoperative characteristics such as the residence time in PACU and the use of analgesic and antiemetic drugs were not significantly different in the two groups.

**Table 2:** Nausea and vomiting outcomes and postoperative characteristics.

**Outcomes/postoperative characteristics**	**DEX + AZA (*n* = 84)**	**AZA (*n* = 84)**	***P*** **-value**
**PONV, no. (%)**			
PACU	0	0	–
PACU-6 h	15 (18)	16 (19)	0.842^a^
6–24 h	20 (24)	24 (29)	0.483^a^
0–24 h	30 (36)	32 (38)	0.749^a^
**Nausea, no. (%)**			
PACU	0	0	–
PACU-6 h	9 (11)	12 (14)	0.484^a^
6–24 h	13 (15)	12 (14)	0.828^a^
**Vomiting, no. (%)**			
PACU	0	0	–
PACU-6 h	9 (11)	5 (6)	0.264^a^
6–24 h	12 (14)	18 (21)	0.227^a^
Use of rescue antiemetics, no. (%)	19 (23)	23 (27)	0.476^a^
Use of rescue analgesics, no. (%)	5 (6)	4 (5)	0.732^a^
Median residence time in PACU (IQR), min	45 (40, 55)	45 (40, 55)	0.391^b^

*DEX, dexmedetomidine; AZA, azasetron; IQR, interquartile range; PONV, postoperative nausea and/or vomiting; PACU, Post-anesthesia Care Unit.*

a *Analyzed using χ^2^ test.*

b *Analyzed using Mann–Whitney U test*.

**Figure 2:**
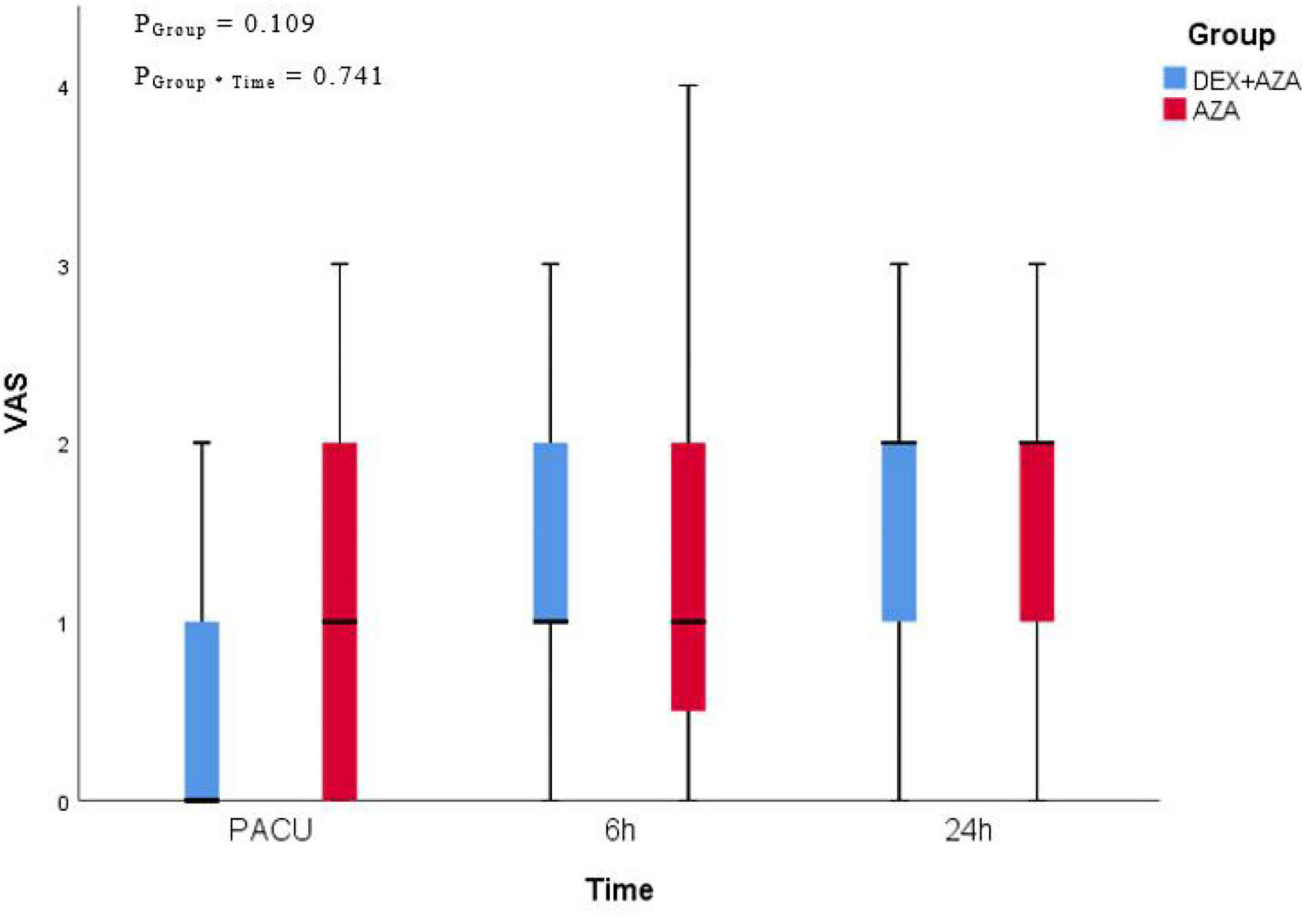
Postoperative pain assessment. We adopted 11-point visual analog scale (VAS) for pain assessment at the time of leaving PACU, 6 and 24 hours after thyroidectomy. VAS presented as median (interquartile range). In the DEX + AZA group (*n* = 84), patients received dexmedetomidine 0.5 μg kg^−1^ for 10 min before anesthesia, and then the infusion rate was held at 0.1 μg kg^−1^ h^−1^ until the completion of operation. In the AZA group (*n* = 84), patients received an equal volume of saline with same pattern as the DEX + AZA group. Patients in both groups were given 10 mg azasetron before the end of surgery. P_Group*Time_ = *P*-value of the group and time interaction obtained by the linear mixed model. DEX + AZA, dexmedetomidine + azasetron; AZA, azasetron.

**Figure 3:**
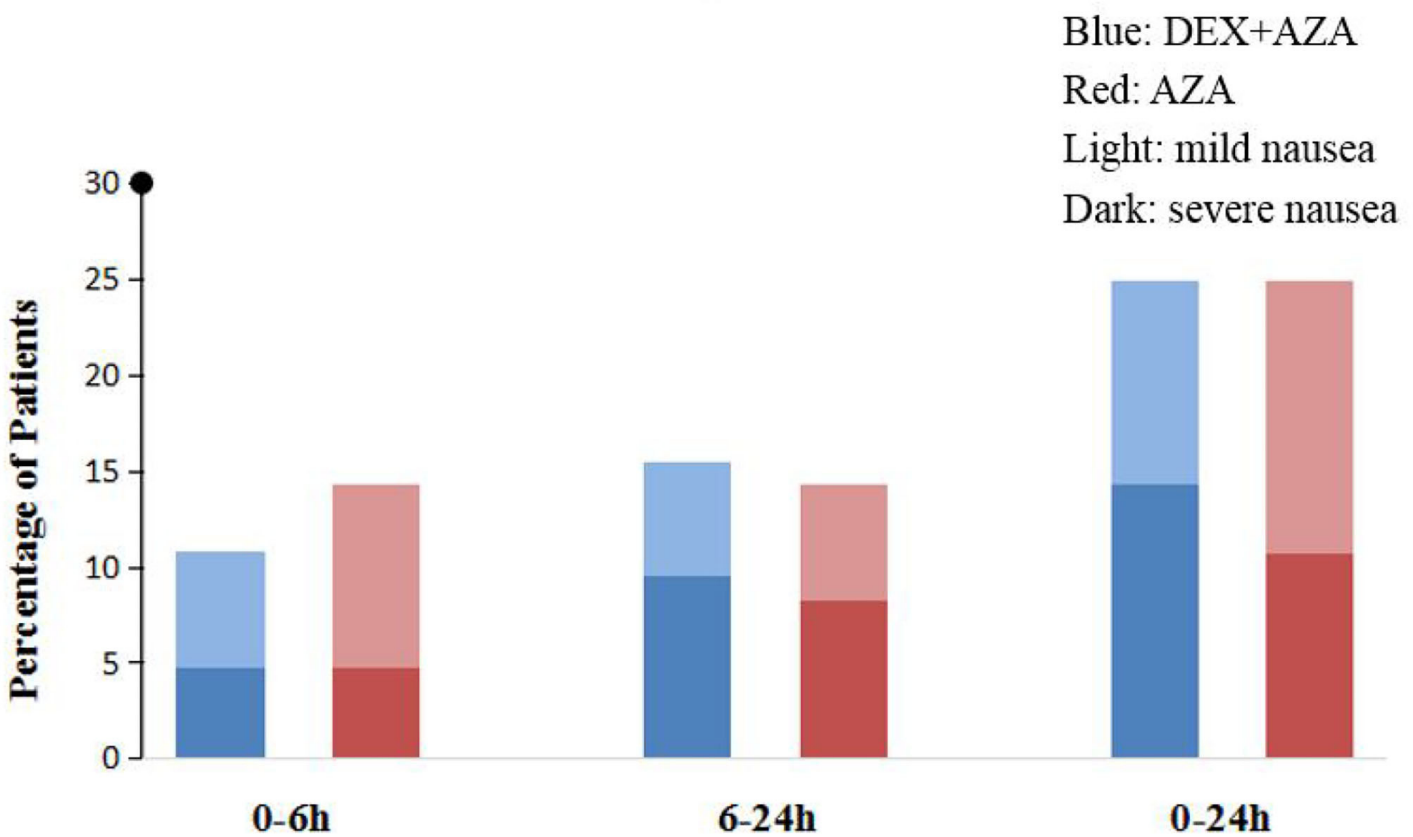
Postoperative nausea intensity assessment. We adopted 11-point numerical rating scale (NRS) to assess nausea severity (0, no nausea; 10, worst nausea imaginable). Mild nausea is defined as NRS score from 1 to 3, whereas severe nausea is >3. In the DEX + AZA group (*n* = 84), patients received dexmedetomidine 0.5 μg kg^−1^ for 10 min before anesthesia, and then the infusion rate was held at 0.1 μg kg^−1^ h^−1^ until the completion of operation. In the AZA group (*n* = 84), patients received an equal volume of saline with same pattern as the DEX + AZA group. Patients in both groups were given 10 mg azasetron before the end of surgery. DEX + AZA, dexmedetomidine+ azasetron; AZA, azasetron; Light, mild nausea; Dark, severe nausea.

Throughout the study period, the VAS scores were not significantly different at any time points between the two groups (Figure 2). Postoperative nausea occurred in 21 patients in each group. The severity of nausea was similar in both groups 24 h after surgery; severe nausea (NRS score, >3) occurred in 9 patients (43%) in the AZA group and 12 patients (57%) in the DEX+AZA group (relative risk, 1.33; 95% CI, 0.72–2.50; *P* = 0.355) (Figure 3). No serious adverse events were observed during the study. The main intraoperative adverse events were hypotension and bradycardia, and the rate did not differ between the two groups (Table 3).

**Table 3:** Comparison of perioperative adverse events.

**Variables**	**DEX + AZA (*n* = 84)**	**AZA (*n* = 84)**	***P*** **-value**
**Adverse events during operation**			
Hypotension, no. (%)	2 (2)	2 (2)	–
Bradycardia, no. (%)	6 (7)	8 (10)	0.577^a^
**Adverse events in the PACU**			
Agitation, no. (%)	1 (1)	3 (4)	0.613^b^
Over sedation, no. (%)	6 (7)	3 (4)	0.493^b^

*DEX, dexmedetomidine; AZA, azasetron; PACU, Post-anesthesia Care Unit.*

a *Analyzed using χ^2^ test.*

b *Analyzed using continuity correction χ^2^ test.*

## Discussion

The results in this randomized, double-blind trial suggest that intra-operative dexmedetomidine combined with azasetron did not significantly decrease the rate of 24-h PONV compared with azasetron alone.

The mechanism of PONV is very complex, and the trauma caused by surgery, inflammation, visceral stimulation, pain, hypoxia, and hypotension are the main stimulating factors (14). When the stimulus reaches the vomiting center *via* the afferent nerve, dopamine, histamine, serotonin type 3 (5-HT3), tachykinin 1 (NK1), or muscarinic receptors are activated to trigger the vomiting response (15). Moreover, relevant studies have reported that female sex, non-smoker, history of PONV, and use of postoperative opioids are high-risk factors for PONV, and the occurrence of PONV is also related to the type of surgery and intraoperative anesthesia management (16). The incidence of PONV varies greatly among different types of surgery, such as otolaryngology, gynecological, and endoscopic surgeries with relatively high incidence of PONV. In the maintenance of intraoperative anesthesia, inhalation of anesthetic drugs can easily cause PONV compared with total intravenous anesthesia (TIVA) (17, 18).

Geng et al. (13) conducted a study to investigate the effect of adjuvant dexmedetomidine on PONV during gynecological laparoscopic surgery. The results indicate that intraoperative dexmedetomidine (0.5 μg kg^−1^ loading dose followed by 0.1 μg kg^−1^ h^−1^ infusion until the end of surgery) can reduce the incidence of postoperative nausea 2 h after surgery. Song et al. (19) found that, in orthopedic surgery, adding 10 μg kg^−1^ dexmedetomidine to a fentanyl-based PCA drug mixture improved the frequency and intensity of severe postoperative nausea in patients with high-risk factors of PONV. However, the mechanism of the antiemetic function of dexmedetomidine is still unknown. Possible mechanisms to reduce PONV by dexmedetomidine include the following: dexmedetomidine has sedative and analgesic effects and can reduce the dose of narcotic drugs and opioids. It can also inhibit the excitability of the sympathetic nerve and reduce the release of catecholamine, because catecholamine may be a contributing factor of PONV (5).

This trial aimed to clarify the effect of dexmedetomidine combined with azasetron on PONV. Unfortunately, it fails to further drop the occurrence of PONV, but the gross incidence of PONV in the 2 groups was not as high as reported in previous articles. This may be caused by the fact that we have adopted a multimodal prophylactic approach to prevent PONV, including TIVA with propofol, no inhalation anesthesia was used and no postoperative opioid used, which reduces the baseline risk of PONV.

There may be several reasons for the differences in the outcomes between the present study and the previous articles. First, a multimodal prophylactic treatment was used to accelerate the patient's recovery after surgery, but too many antiemetic factors may have masked the effect of dexmedetomidine on PONV. Second, due to the sedative and analgesic properties of dexmedetomidine, the amount of intraoperative remifentanil in the experimental group was significantly reduced compared with the control group. However, many studies have reported that remifentanil has a short half-life and quick elimination, and there is no effect on PONV (20–22). Therefore, although dexmedetomidine reduced the dose of intraoperative remifentanil, it fails to drop the occurrence of PONV. Third, Aouad et al. reported that 1, 0.5, and 0.25 μg kg^−1^ dose of dexmedetomidine had no significant difference in the occurrence of PONV, but they cause dose-dependent hypotension (23). For decreasing the incidence of perioperative adverse events and promoting postoperative recovery, the selected dose of dexmedetomidine (0.5 μg kg^−1^ loading dose followed by 0.1 μg kg^−1^ h^−1^ infusion) may be relatively small, which may affect the antiemetic effect of dexmedetomidine. Finally, dexmedetomidine may not decrease the incidence of PONV.

There are several limitations in this study. First, our trial did not adopt a multicenter study method, which may make our results not universal. Second, only one dose of dexmedetomidine was adopted in this trial, and this dose of dexmedetomidine combined with azasetron did not produce significant effects in patients undergoing ambulatory thyroidectomy. Third, we included patients with ambulatory thyroidectomy in our study, and traditional procedures such as cholecystectomy and endoscopic surgery with a high incidence of PONV were not selected. In future studies, we can choose different types of surgery to compare the incidence of PONV.

In summary, we find that intraoperative dexmedetomidine combined with azasetron did not significantly decrease the incidence of 24-h PONV compared with azasetron alone in adult patients undergoing ambulatory thyroidectomy.

## Data Availability Statement

The original contributions presented in the study are included in the article/Supplementary Material, further inquiries can be directed to the corresponding authors.

## Ethics Statement

The studies involving human participants were reviewed and approved by Ethics Committee of The First Affiliated Hospital of Anhui Medical University. The patients/participants provided their written informed consent to participate in this study.

## Author Contributions

CX and YL: study design. CX: ethics approval and registration. CX, CZ, and HS: patient recruitment. CX and CZ: data collection. CX and HS: data analysis. CX, CZ, and YL: drafting. All authors contributed to the article and approved the submitted version.

## Funding

This work was partially supported by the National Natural Science Foundation of China (No. 81770295).

## Conflict of Interest

The authors declare that the research was conducted in the absence of any commercial or financial relationships that could be construed as a potential conflict of interest.

## Publisher's Note

All claims expressed in this article are solely those of the authors and do not necessarily represent those of their affiliated organizations, or those of the publisher, the editors and the reviewers. Any product that may be evaluated in this article, or claim that may be made by its manufacturer, is not guaranteed or endorsed by the publisher.
